# Public perception on active aging after COVID-19: an unsupervised machine learning analysis of 44,343 posts

**DOI:** 10.3389/fpubh.2024.1329704

**Published:** 2024-03-07

**Authors:** Peipei Chen, Yuwei Jin, Xinfang Ma, Yan Lin

**Affiliations:** School of Foreign Language Studies, Wenzhou Medical University, Wenzhou, Zhejiang, China

**Keywords:** active aging, public perception, topic modeling, BERTopic, policy making

## Abstract

**Introduction:**

To analyze public perceptions of active aging in China on mainstream social media platforms to determine whether the “14th Five Year Plan for the Development of the Aging Career and Older Adult Care System” issued by the CPC in 2022 has fully addressed public needs.

**Methods:**

The original tweets posted on Weibo between January 1, 2020, and June 30, 2022, containing the words “aging” or “old age” were extracted. A bidirectional encoder representation from transformers (BERT)-based model was used to generate themes related to this perception. A qualitative thematic analysis and an independent review of the theme labels were conducted by the researchers.

**Results:**

The findings indicate that public perceptions revolved around four themes: (1) health prevention and protection, (2) convenient living environments, (3) cognitive health and social integration, and (4) protecting the rights and interests of the older adult.

**Discussion:**

Our study found that although the Plan aligns with most of these themes, it lacks clear planning for financial security and marital life.

## 1 Introduction

According to China's seventh population census in 2020, there are now 264 million older Chinese adults, representing 18.7% of the population. By 2025, the population of China's older adults is expected to exceed 500 million, representing 38.81% of the total population, with over 10% of the population being over the age of 80 (1). The government, society, and individuals all face the crucial task of mitigating the negative impacts of an aging society and enhancing the social value of older adults. To address this challenge, the World Health Organization introduced the concept of “active aging” as a means to optimize health, social participation, and security to enhance the quality of life as people age (2). Active aging emphasizes not only the absence of disease or disability but also the ability to maintain physical and mental wellbeing, engage in social activities, and continue to contribute to society (3, 4). Unlike previous aging ideals such as “healthy aging,” active aging recognizes the pivotal role that social involvement plays in the lives of older individuals as they age (5).

In this context, the CPC Central Committee and the State Council released “The 14th Five-Year Plan for the Development of the National Aging Career and Older Adult Care System” (2022), which provides guidelines for actively responding to aging populations. These priorities include (1) improving older adult care services; (2) improving support systems; (3) developing multiple business models; (4) strengthening security capabilities; and (5) creating an age-friendly environment.

However, the COVID-19 pandemic has significantly altered public perceptions of aging, particularly among older adults, causing many of them to reevaluate their understanding of a fulfilling life. As a result of social distance, many older adults have become more dependent on their families, making communal living a difficult choice (6). Furthermore, social media has become a valuable tool for seniors to combat feelings of isolation and a lack of social support (7). In light of this, older adults are increasingly embracing a digital lifestyle and adapting their views on aging. To gain a better understanding of the public's perception of active aging, a study was conducted analyzing conversations about aging on mainstream Chinese social media platforms. Through BERT-based text topic mining analysis and qualitative thematic analysis, we identified key concerns related to active aging as discussed by the Chinese public. By comparing them to the priorities set forth in the Plan, we aim to improve the effectiveness of active aging policies by bridging the gap between public perception and government initiatives.

## 2 Materials and methods

The data for this study is collected from Sina Weibo, one of the most popular social media platforms in China with over 500 million registered users and around 200 million monthly active users in 2023. Tweets containing the following keywords (“aging” or “old age” or “active aging” or “healthy aging”) between January 1, 2020 and June 30, 2022. In order to focus on original tweets posted by individuals, we pre-processed the data by removing duplicate comments, noisy data, organizational tweets, and irrelevant comments. The final dataset for this study was 44,343.

In this study, we utilized a bidirectional encoder representation from transformers (BERT)-based model. A widely recognized state-of-the-art deep machine learning approach for natural language processing (NLP), BERT employs unsupervised masked language models (MLM) and the next sentence prediction (NSP) for text deep pretraining and fine-tuning (8). This algorithm has demonstrated impressive performance in multiple NLP tasks, including machine reading comprehension and sentiment analysis (9). MLM involves randomly masking or replacing certain words or phrases in a sentence, and predicting the words that are missing. NSP, on the other hand, trains the model to determine the contextual relationship between two randomly presented sentences. In contrast to traditional word embedding techniques such as Word2Vec or Glove, BERT takes into account each word's contextual significance and the varying meanings of expressions in different contexts. As its name suggests, BERTopic is a topic modeling algorithm that generates topic clusters using BERT word vectors, Transformer, and c-TF-IDF (10). In this method, the topics are explained clearly while preserving the important words in the topic descriptions. We selected BERTopic for our study because existing studies have demonstrated BERTopic to be significantly more effective than traditional topic models such as LDA when it comes to depth of topic words, specialization, clear-inter-word relationships, and retention of contextual semantic information (11, 12).

For this study, we used the Chinese pre-training model provided by BERT and integrated it into our own text classification system. The BERT model was called using the BERT-as-service open-source service provided by Tencent AI Lab. This allowed us to convert microblog posts into 768-dimensional sentence vectors and extract the topics for the analysis of the later text. The researchers conducted a qualitative thematic analysis, independently reviewing labels and themes. They familiarized themselves with keywords and sample tweets, customized and refined themes, and sought consensus on coding through group discussions.

## 3 Results

Data were collected from 287,942 original tweets containing keywords such as “aging” or “old age” or “healthy aging” or “active aging” posted on Weibo between January 1, 2020 and June 30, 2022. After removing duplicate posts as well as tweets by organizations, the study included a final total of 44,343 posts.

**Figure 1:**
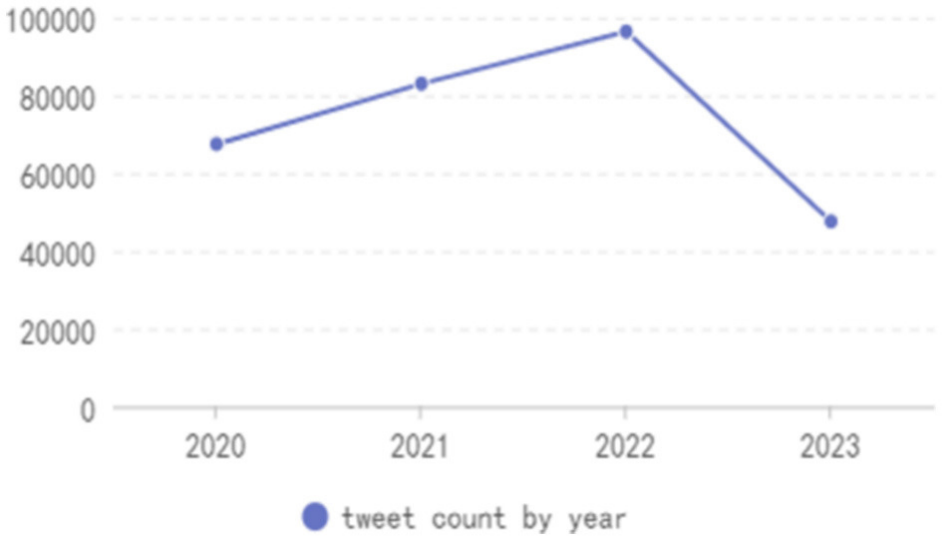
Tweet count by year.

Figure 1 displays the annual distribution of posts, revealing a consistent focus on “aging” and related subjects on Weibo throughout the years 2020 to 2022. This phenomenon aligns with the present circumstances, considering the disproportionate impact of COVID-19 on older adult populations since its emergence in December 2019.

The current study generated 20 prominent topics related to the public views regarding health and aging issues (Table 1). The most prominent topic, Topic 1, focused on COVID-19 confirmation and infection. To analyze the data, the researchers manually grouped the topics into four main themes using thematic analysis. This process involved individual familiarization with the data, theme generation and review, and naming of the themes during offline meetings (13, 14).

**Table 1:** Themes related to public perceptions of active aging, along with the respective topics and sample tweets (*N* = 44,343).

**Theme and topic (key words)**	**Sample tweets**	**Number of tweets, *n* (%)**
**Theme 1: Health prevention and protection**		
Topic 1. COVID-19 prevention (*confirm, infection, death, inoculation, immunization, fever, rescue, and vulnerable*)	“There are many older adults, and there are many severe cases. It's really hard. The city that is aging first is too vulnerable in the face of the epidemic.”	5,377 (12.13)
Topic 5. Maintaining sleep quality *(Sleeplessness, morning, sleep, five o'clock, intake, sleep, exercise, obesity, insomnia, shallow sleep, dreaming, and waking)*	“I have been experiencing insomnia for several days now. I can't fall asleep at night, and I wake up early in the morning. My body is aging rapidly.”	1,098 (2.5)
Topic 3. Cancer prevention (*Chronic, Killer, Malignant, Tumor, Killer, Dangerous, Removal Malignant, Tumor, Vertigo, Brain, Tumor, and Watchfulness*)	“'Day 449 of my father's fight against cancer' |Returned the senior phone I bought for my father. As one ages, they become increasingly reliant on others, which many young people have yet to realize. The number of older adults living alone is increasing, and their needs for services are becoming more profound. Hopefully, every older adult can be treated with kindness.”	698 (1.6)
Topic 7. Keeping fit (*Keeping fit, health management, nutritional supports, tonifying the kidneys, Chinese medicine, curing the disease, preventive care, promoting blood circulation, and removing blood stasis*)	“As you age, your physical functions decline. My thoracic vertebrae are severely misaligned. When I arrived, I couldn't bow my head or turn around, and I dare not take deep breaths. But after receiving 12 acupuncture treatments, now I can jump around energetically. Truly amazing, the miracle of traditional Chinese medicine.”	552 (1.3)
Topic 10. Hearing impairment *(Deafness, hearing, fitting, wearing, hearing aids, hearing impairment, hearing loss, and fading)*	“Many older people do suffer from hearing loss, and their lives become quite inconvenient as they age. It's important to genuinely care about the health of our older adult family members! Older people face challenges in communicating with their loved ones due to their hearing impairments.”	512 (1.2)
Topic 9. Maintaining bone health *(Rheumatism, osteoporosis, artificial joints, joint pain, lower back pain, pain, rheumatoid, and arthritis)*	“My finger joints hurt, my neck hurts, and I even experience lower back pain when I catch a cold. Even in the summer, I feel more comfortable wearing more layers. When I retire, I should move to a tropical area for my old age.”	445 (1.0)
Topic 4. Maintaining healthy skin (*face, sagging, sunken, sagging, wrinkles, skin, care, sagging, skin, skin, elasticity, pale, and skin*)	“White hair is increasing, wrinkles are becoming more obvious, I dare not look in the mirror after washing my face every day. The reflection in the mirror, I don't even like myself.”	240 (0.5)
**Theme 2: A safe and convenient living environment**		
Topic 19. Living convenience *(Evening, next to, go out, to the supermarket, convenience, suitable for the older adult, at home service, eating and living, and on time)*	“In our village, there is a significant aging population among party members, and the meeting room is located on the third floor. It is inconvenient for older adult with physical decline to go up and down the stairs. Therefore, volunteer party members from Nanhui Village help support the older adult to attend meetings in the conference room!”	1,667 (3.8)
Topic 17. Prevention of home safety hazards *(Downstairs, addition, proprietary, safety, call for help, handrail, suitable for the older adult, heating system, safety hazards, suitable for the older adult, personal privacy, and appliances)*	“This is a difficult problem with no easy solution. Everyone will grow old one day, and caring for the older adult is a matter of concern for all of us. With the trend of an aging society, a profession called “bathing assistants” has emerged in some big cities in recent years. Their job is to help the older adult bathe. However, the safety risks of bathing for the older adult also need to be considered.”	596 (1.3)
Topic 12. Wandering prevention *(Neighborhood, safety, wander, living alone, wandering prevention, research, wandering, consciousness, and loneliness)*	“Through the 5.9 No-Wandering Day Volunteers' Program, many people have become aware of Alzheimer's Disease, and have learned that when seniors with yellow bracelets seek help, they should get home safely!”	276 (0.6)
Topic 18. Eating convenience *(Ordering takeout, in trouble, dining, consumption, dining, food delivery, food preparation, eating, and three meals a day)*	“As I age, my stomach cannot handle the food cooked by young people. Moreover, my lunch and dinner time is at 11 AM and 4 PM, which doesn't align with the timing of young people. I can't disturb them to cook a separate meal just for me.”	286 (0.6)
**Theme 3: Cognitive health and social integration**		
Topic 13. Retirement life *(Delayed, retirement payment, contribution, retirement, delayed retirement, early retirement, epidemic, and procrastinated)*	“I strongly oppose extending the retirement age. Working for 3 h in the morning and afternoon, and commuting in packed subway trains every day is already exhausting for someone who is 45 years old. How can we expect them to continue working until they are 65?”	1,501 (3.4)
Topic 15. Senior citizens' university *(Senior college, older adult learning, education, teaching, older adult life, community college, and social life)*	“At the senior citizens' university, we sometimes organize charity performances and have private gatherings for karaoke, which greatly enriches my senior life. But you have to sign up quickly, otherwise, the spots will easily be taken.”	567 (1.3)
Topic 16. Social integration *(Older adult, friendly, healthy, neighborhood, widowed older adult, service, social life, friends, and wellbeing)*	“There are also social needs, which many people who don't buy groceries fail to analyze. When people get old, it is impossible for them to stay at home every day. Going out to buy groceries is one of the necessary activities.”	1,263 (2.8)
Topic 11. Maintaining cognitive and mental health *(Alzheimer's, dementia, memory, aging brain, Alzheimer's, aging, research, memory loss, and brain health)*	“There is talk in the group that there is an old grandma who is very skilled at playing esports. I support the old grandma playing esports for several reasons: firstly, it can help slow down the onset of dementia in old age; secondly, it is very suitable for the needs of an aging society; thirdly, with the use of a brain-computer interface headset, even older adult can play, making it very suitable for nursing homes.”	346 (0.8)
**Theme 4: The rights and interests of the older adult**		
Topic 6. Fertility policy adjustment (*Two-child policy, open up, fertility, three-child, having two children, demographic crisis, family planning, and birth control*)	“What's the point of having more children? The cost of living is too high in today's society, and it's better not to have children because the stress is too great.”	2,309 (5.2)
Topic 20. Medical companion *(Medical escort, medical advice, health management, medication, post-surgery care, and medical accompaniment)*	“Many older adults who come to seek medical treatment alone do not know how to use mobile applications and self-service machines in hospitals. They have nobody to help them, so they are unable to get a queue number.”	859 (1.9)
Topic 2. Scam prevention (*Fraud, scammers, scam prevention, fight scam, scam awareness, fraud, fraudulent activity, and scam awareness*)	“With the increasingly aging population in China, the number of older adults is constantly growing. Some unscrupulous individuals take advantage of this situation to get close to the older adult, gain their trust, and then carry out various scams targeting the older adults.”	632 (1.4)
Topic 8. Marriage (*Divorce, cheating, marriage, mutual cause, knot, binding, expenses, anchor, feed the family, and marriage*)	“Times have changed. With the awakening of women's consciousness & economic independence, more and more women have higher expectations for the quality of marriage. The increasing divorce rates year by year also indicate that women have lower tolerance for low-quality marriages.”	537 (1.2)
Topic 14. Inheritance and post mortem arrangements *(End of life, hospice, terminal, estate, inheritance, dependence, legacy, and funeral)*	“End-of-life care and population aging are global challenges. It is difficult for vibrant young people to imagine what it feels like to wait for death.”	246 (0.6)

According to Table 1, theme 1 (health prevention and protection) holds the highest prevalence, accounting for 20.23% of the total. Among the topics within this theme, topic 1 (COVID-19 prevention) is the most prominent, representing 12.13% of the total, while topic 4 (maintaining healthy skin) has the lowest share at 0.5%. The second most prominent theme is Theme 4 (the rights and interests of the older adult), comprising 10.3% of the total. Within this theme, topic 6 (fertility policy adjustment) stands out the most prominent, accounting for 5.2%, while topic 14 (inheritance and post mortem arrangements) ranks the least prominent at 0.6%. Theme 2 (a safe and convenient living environment) has a prevalence of 6.3%, with topic 19 (living convenience) being the most prominent at 3.8% and topic 18/12 (eating convenience, wandering prevention) both the least prominent at 0.6%. Theme 3 (cognitive health and social integration) has a prevalence of 8.3%. Topic 13 (retirement life) emerges as the most prominent topic within this theme, representing 3.4% of the total. Conversely, topic 11 (maintaining cognitive and mental health) ranks the least prominent, accounting for only 0.8%.

In addition to topic 1, the survey also identified three other prominent topics that have sparked considerable public discussions. These topics include topic 16 (fertility policy adjustment, accounting for 5.2% of the discussion), topic 19 (living convenience, accounting for 3.8% of the discussion), and topic 13 (retirement life, accounting for 3.4% of the discussion). Interestingly, topics that were traditionally considered to be of greater concern to Chinese seniors, such as topic 18 (eating convenience), received relatively less attention in this survey, only accounting for 0.8% of the discussion.

## 4 Discussion

**Figure 2:**
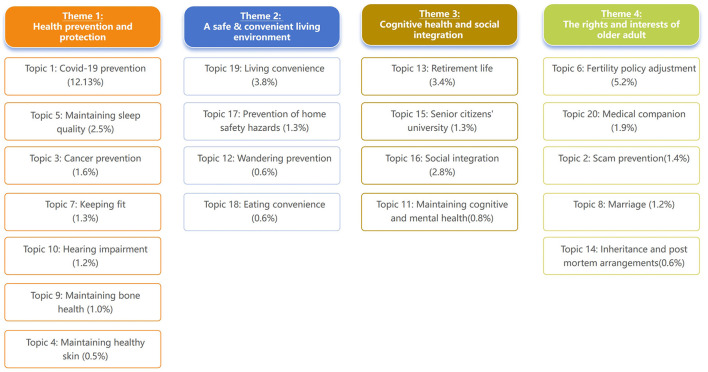
Classification of themes and topics uncovered in the current study (percentages represent the prevalence of the individual topics).

In our study, unsupervised machine learning was employed to analyze a total of 44,343 posts from a major social media platform. And arising 20 broad topics were further categorized into four themes through thematic analysis. These four themes are health prevention and protection, a safe and convenient living environment, cognitive health and social integration; and the rights and interests of the older adult (as shown in Figure 2).

Health prevention and protection (theme 1) is of utmost importance for the older adult population, especially during and after the COVID-19 epidemic. It is worth noting that the average age of COVID-19-related deaths in China was 80.3 years, with a significant proportion of the population being 65 years or older (90.1%), and 80 years or older (56.5%) (15). This study, conducted from January 2020 to June 2023, observed a consistent public interest in COVID-19 prevention (Topic 1), which aligns with the current social conditions. Additionally, the remaining topics within this theme emphasize the importance of enabling older individuals to maintain normal physical function, which is crucial for their active engagement in society as outlined in the Plan. This includes addressing various aspects of health, such as sleep quality (Topic 5), cancer prevention (Topic 3), hearing impairment (Topic 10), bone health (Topic 9), and skin health (Topic 4). These areas play a significant role in older people's sense of self-worth and independence (16).

Specifically, sleep quality (Topic 5) and cancer prevention (Topic 3) have received substantial attention. Previous studies have highlighted that poor sleep quality in older adults can have detrimental effects on cognitive function (17), increase the risk of neurological and psychiatric disease (18), and contribute to social isolation and feelings of loneliness (19). Notably, sleep disorders are highly prevalent among Chinese older adults in both rural and urban areas (20, 21). As to cancer prevention, it has always been a top priority for the Chinese government to promote a healthy and active aging society. To reduce the burden of cancer, collaborative efforts from various sectors have been implemented to mitigate factors such as environmental pollution, tobacco use, occupational carcinogens, excessive alcohol consumption, dietary deficiencies, and obesity (22, 23).

Themes 2 focuses on promoting a safe and convenient living environment. This aligns perfectly with the goals of creating an active and livable aging environment. Living and eating convenience (Topic 19 and Topic 18) are in line with the requirements of the Plan. Additionally, the prevention of wandering and hazards at home (Topic 12 and Topic 17) is essential for family care, especially considering that older adults are more susceptible to issues such as declining muscle strength and reduced adaptability to environmental changes (24). However, it is worth noting that Chinese households generally lack the necessary assistive technologies (25) to effectively prevent falls, wondering, and accidents (26, 27).

The importance of cognitive health and social integration (theme 3) for active aging cannot be overstated enough. An active and healthy social integration (Topic 16) and a well-planned retirement life (Topic 13) contribute to good physical, psychological, and cognitive wellbeing in seniors (28, 29). There is increasing public awareness in this area, as it has been proven to enhance the overall wellbeing and life expectancy of older adults (30). However, contemporary older adults in China faces a new set of challenges that are distinct from traditional ones. These challenges include the consequences of the two-child policy and the substantial financial losses caused by financial fraud (31). Senior citizens' universities (Topic 15) have also become a hot topic of discussion on social media. With over 70,000 senior colleges in China, these institutions provide gerontological education and cater to the diverse spiritual and cultural needs of the older adult population. They offer a wide range of educational resources, alleviating the pressure of population aging and achieving the goal of “learning and enjoyment for the older adult” (32) as set in the Plan for active aging.

The Plan focuses on improving the support systems to safeguard the rights and interests of the older adult (theme 4). It acknowledges the importance of healthcare services (Topic 20 Medical companion) in promoting healthy aging, such as establishing family hospital beds and facilitating home visits. However, our analysis reveals that the public's needs encompass a broader range of concerns beyond medical care. These include adapting to the negative impact brought by fertility policy adjustment (topic 6), ensuring financial security (topic 2 scam prevention), addressing marital life issues (topic 8 marriage), and managing inheritance and post-mortem arrangements (topic 14).

The Chinese government has recently relaxed its birth control policy, allowing couples to have two or three children instead of the previous limit of one. This adjustment reflects a change in their fertility policy. However, our survey has revealed a significant amount of attention on social media (2,309 posts, 5.2%) toward the negative impact of this policy change on older adults, yet it has not been adequately addressed in the Plan. Due to Confucian family culture, Chinese older adults often take on the role of providing intergenerational care for their grandchildren, a practice that is nearly 10 times more prevalent compared to South Korea (33). While some studies suggest that intergenerational caregiving can have positive effects on older adults' physical and mental health (34, 35), it is generally acknowledged that excessive intergenerational caregiving can result in increased physical exhaustion (36), a higher risk of mental and physical ailments (37), and a decrease in socialization opportunities (38).

Thus, we believe that the Chinese government should address the societal perception that raising a second child is a burden, particularly for grandparents, by transforming child-rearing support into a welfare system. To achieve this, they can draw inspiration from European countries' childcare welfare systems, such as the UK's “Specified Adult Childcare credits,” “Major New Extensions of Shared Parental Leave,” and “Tax-free Children” (39). Additionally, the government should increase fiscal investment and lower the age limit for children entering kindergartens (40). By implementing these policies, grandparents would receive necessary support, thereby reducing their long-term responsibilities in caring for their grandchildren and alleviating the pressure on families.

Moreover, the government should address two other pressing issues concerning the wellbeing of older individuals: their financial security (Topic 2) and their marriages (Topic 8). In China, Internet scams have become the main form of fraud, with older adults being the primary victims of this crime due to their limited ability to discern online information and lack of immediate assistance (41, 42). Surprisingly, the Plan appears to overlook any specific initiatives aimed at safeguarding the financial assets of older people. Existing research indicates a direct correlation between the level of family contact, the prevalence of depression, and the vulnerability to deception among older adults (31, 42). Therefore, it is crucial for families to enhance their companionship with relatives of older adults, for the government to promote financial literacy and anti-fraud measures to enhance older people's fraud awareness, and for financial institutions to develop tailored financial products that help the older adult rationally plan for retirement finances (42).

Furthermore, older adults often encounter intricate challenges in their marital lives due to various factors such as financial constraints and social pressures. The loss of a spouse, known as widowhood, is a common struggle that can result in physical health deterioration, increased mortality rates, and psychological symptoms like depression and anxiety (43). Remarriage can be an effective way to restore the benefits of a marital relationship (44). However, in Chinese society, remarriages are often influenced by financial considerations, societal opinions, age-related issues, and inheritance matters. As older individuals seek solutions to address their feelings of spiritual emptiness and inheritance problems, non-traditional forms of remarriages, such as “cohabitation without marriage” and “contract marriage,” emerged. This social phenomenon has generated discussions on mainstream social media in China, yet there are currently no specific plans or measures in place to safeguard older people's marriages.

Given the increasing prevalence of non-marital cohabitation, it would be wise for the Chinese government to enhance the existing marriage law system by incorporating elements from the legislative model of the foreign non-marital cohabitation system (45). Additionally, the establishment of a contractual system for non-marital cohabitation (46) could be put in place. By doing so, a comprehensive legal framework can be established to address these dynamics and provide a foundation for safeguarding the interests of both partners and their offspring, while also ensuring the protection of the more vulnerable party involved.

## 5 Conclusion

In conclusion, this study analyzed the key public perceptions of active aging, as represented by Weibo users from January 1, 2020, to June 30, 2022, to see if the “14th Five Year Plan for the Development of the Aging Career and Older Adult Care System” issued by the CPC in 2022 has fully addressed public needs. The research findings show that the Pan for active aging appears to align well with the future objectives and work priorities, as it covers many prominent topics of public concern. However, there are certain topics that have not been adequately addressed in the Plan, such as fertility policy adjustment, financial security, social integration, and marital life. These topics have gained public attention, indicating that the concerns of older adults in China have expanded beyond basic healthcare and daily life needs. They now encompass a broader and more complex range of psychological and social needs. In line with these findings, it is important for future government efforts to prioritize not only the existing topics in the Plan but also consider additional areas such as fertility policy adjustment, financial security, marital life, and social integration. These findings may provide the government and organizations with valuable insights for better active-aging initiatives.

## 6 Limitations and future work

However, it is important to consider the limitations of this study. Firstly, the findings may not be representative of the entire population as they primarily reflect the views of the Chinese public expressed on Weibo, a major social platform, but not the only one. It is important to include perspectives from other platforms such as TikTok, an emerging micro-video sharing app that has gained popularity among older adults. Future research should aim to capture and analyze the voices of the aging population on TikTok thus providing a more comprehensive understanding of their perceptions. Secondly, participation on Weibo requires a certain level of literacy, which means that illiterate older adults may be excluded from expressing their opinions on this social platform. To overcome this limitation, future researchers could consider incorporating methods such as surveys and interviews to obtain the perceptions of older adults who may not be active on social media platforms. By combining multiple data sources, researchers can strive for multiple verifications of their research findings. Last, it is worth noting that some tweets seemed to be advertisements or product placements, which may have influenced the accuracy of the findings. Although we excluded organizational tweets, we couldn't exclude individual tweets promoting products for commercial gain. Future research could explore additional techniques, such as Zipf's law (47) to reduce statistic noise and improve accuracy. Additionally, novel topic modeling approaches like GPT 4.0 and WuDao 2.0 could be employed to further improve the analysis.

## Data availability statement

The original contributions presented in the study are included in the article/supplementary material, further inquiries can be directed to the corresponding author.

## Author contributions

PC: Writing – original draft, Writing – review & editing. YJ: Conceptualization, Investigation, Writing – review & editing. XM: Software, Writing – review & editing. YL: Writing – review & editing.
